# Supplementary material for: Global hypo-methylation in a proportion of glioblastoma enriched for an astrocytic signature is associated with increased invasion and altered immune landscape
Source: eLife. 2022 Nov 22;11:e77335. doi: 10.7554/eLife.77335 (PMC9681209; doi:10.7554/eLife.77335)
Supplement: Figure 2—source data 1. [file elife-77335-fig2-data1.zip › Figure_2_source_data_1/Figure_2A/homerResults/motif7.similar.html]

motif7

## Information for motif7

A
T
G
C
A
G
T
C
G
T
A
C
A
G
C
T
C
A
T
G
A
G
T
C
A
G
T
C
G
A
C
T
A
T
G
C
A
G
C
T
A
T
G
C
A
T
G
C
G
T
A
C
C
G
A
T
T
A
C
G
A
T
G
C
A
G
T
C
A
T
G
C
G
A
T
C
G
A
T
C
  
Reverse Opposite:  

C
T
A
G
C
T
A
G
T
A
C
G
T
C
A
G
T
A
C
G
A
T
G
C
G
C
T
A
A
C
T
G
T
A
C
G
T
A
C
G
T
C
G
A
T
A
C
G
C
T
G
A
T
C
A
G
T
C
A
G
G
T
A
C
T
C
G
A
A
C
T
G
C
T
A
G
T
A
C
G
  

|  |  |
| --- | --- |
| p-value: | 1e-27 |
| log p-value: | -6.257e+01 |
| Information Content per bp: | 1.622 |
| Number of Target Sequences with motif | 28.0 |
| Percentage of Target Sequences with motif | 1.21% |
| Number of Background Sequences with motif | 2.9 |
| Percentage of Background Sequences with motif | 0.08% |
| Average Position of motif in Targets | 106.8 +/- 51.8bp |
| Average Position of motif in Background | 75.7 +/- 42.8bp |
| Strand Bias (log2 ratio + to - strand density) | 0.9 |
| Multiplicity (# of sites on avg that occur together) | 1.00 |
| Motif File: | file (matrix) reverse opposite |

### Similar de novo motifs found

|  |  |  |  |  |  |  |  |
| --- | --- | --- | --- | --- | --- | --- | --- |
| Rank | Match Score | Redundant Motif | P-value | log P-value | % of Targets | % of Background | Motif file |
| 1 | 0.658 | A G T C A G C T A G T C A G T C A G T C A G T C A T G C G T A C G T A C G A T C A G T C A G T C T G C A A T G C A T G C G A T C A G C T G T A C G T A C G T A C | 1e-18 | -41.875738 | 0.73% | 0.03% | motif file (matrix) |
| 2 | 0.614 | C A T G C T A G T A C G T C A G A T G C G C T A C A T G A C T G T A C G C G A T A C T G C T A G A T C G A T C G T C G A | 1e-18 | -41.647817 | 0.90% | 0.07% | motif file (matrix) |
| 3 | 0.624 | C T A G C T A G C T A G T C A G T C A G T C A G C T A G T C A G T C A G T C A G C T A G C T A G T C A G C T A G T C A G C T A G C T A G T C A G T C A G T C A G C T A G C T A G C T A G C T A G C T A G | 1e-1 | -3.622838 | 0.13% | 0.03% | motif file (matrix) |
